# Supplementary material for: A Likelihood Approach for Real-Time Calibration of Stochastic Compartmental Epidemic Models
Source: PLoS Comput Biol. 2017 Jan 17;13(1):e1005257. doi: 10.1371/journal.pcbi.1005257 (PMC5240920; doi:10.1371/journal.pcbi.1005257)
Supplement: S1 File — (TAR.GZ) [file pcbi.1005257.s014.tar.gz › HSPH_Online-SI-Revision/Additions/MSS11/n100-extreme_noisy/n100-extreme_noisy_NEW2_table8.pdf]

1	newly 3 weeks spec	526.162	756.92	{723., 794.}	43.8569	0.15767	newly 3 weeks cumul	2501.43	3180.02	{3128., 3238.}	27.1279	0.1042	newly 5 weeks spec	280.291	410.19	{388., 430.}	46.3445	0.165013	newly 5 weeks cumul	3116.21	4146.7	{4085., 4210.}	33.0689	0.124048	newly 46 weeks cumul	6824.91	5278.24	{5201., 5356.}	22.6621	0.111635
2	newly 3 weeks spec	761.	757.11	{723., 791.}	2.84231	0.0124227	newly 3 weeks cumul	3077.	3106.74	{3041., 3178.}	1.51641	0.00652767	newly 5 weeks spec	441.	420.35	{396., 444.}	5.38549	0.0242663	newly 5 weeks cumul	4068.	4092.34	{4017., 4174.}	1.21731	0.00525055	newly 42 weeks cumul	5247.	5325.27	{5213., 5427.}	1.74976	0.0075042
3	newly 3 weeks spec	277.	211.74	{188., 232.}	23.5596	0.117931	newly 3 weeks cumul	1321.	1046.95	{1005., 1086.}	20.7456	0.101152	newly 5 weeks spec	122.	87.02	{72., 101.}	28.8033	0.150912	newly 5 weeks cumul	1607.	1270.26	{1223., 1318.}	20.9546	0.1023	newly 47 weeks cumul	1853.	1426.41	{1362., 1487.}	23.0216	0.113862
4	newly 3 weeks spec	75.	99.36	{87., 113.}	32.5867	0.12023	newly 3 weeks cumul	495.	565.51	{540., 593.}	14.3212	0.0578075	newly 5 weeks spec	26.	32.68	{25., 41.}	29.4615	0.108238	newly 5 weeks cumul	564.	654.51	{626., 680.}	16.1117	0.064594	newly 51 weeks cumul	589.	699.67	{667., 729.}	18.8132	0.0745136
5	newly 3 weeks spec	383.	522.68	{481., 553.}	36.7258	0.135376	newly 3 weeks cumul	1884.	2390.71	{2324., 2455.}	27.077	0.103976	newly 5 weeks spec	164.	245.54	{221., 271.}	50.0366	0.175109	newly 5 weeks cumul	2324.	2996.54	{2931., 3067.}	29.1592	0.111073	newly 48 weeks cumul	2679.	3527.24	{3438., 3607.}	31.9201	0.120259
6	newly 3 weeks spec	620.	688.89	{654., 719.}	11.1113	0.0454481	newly 3 weeks cumul	2591.	2967.54	{2902., 3020.}	14.5326	0.0588783	newly 5 weeks spec	303.	357.33	{332., 383.}	17.9307	0.0709511	newly 5 weeks cumul	3314.	3823.11	{3757., 3897.}	15.3624	0.0620214	newly 46 weeks cumul	4005.	4743.06	{4670., 4832.}	18.4285	0.0734138
7	newly 3 weeks spec	425.	497.36	{461., 528.}	17.0259	0.0677651	newly 3 weeks cumul	2093.	2220.03	{2162., 2277.}	6.06928	0.0255057	newly 5 weeks spec	238.	246.61	{222., 271.}	6.65966	0.0280378	newly 5 weeks cumul	2658.	2818.15	{2755., 2892.}	6.02521	0.0253291	newly 44 weeks cumul	3121.	3391.56	{3303., 3478.}	8.66902	0.0360143
8	newly 3 weeks spec	611.	687.75	{650., 725.}	12.5614	0.0510343	newly 3 weeks cumul	2634.	2966.61	{2907., 3032.}	12.6276	0.0515836	newly 5 weeks spec	315.	359.2	{334., 388.}	14.1524	0.0568617	newly 5 weeks cumul	3371.	3824.69	{3757., 3894.}	13.4586	0.0547915	newly 46 weeks cumul	4133.	4749.41	{4655., 4829.}	14.9143	0.0603311
9	newly 3 weeks spec	509.	690.22	{658., 725.}	35.6031	0.13196	newly 3 weeks cumul	2442.	3043.44	{2992., 3095.}	24.629	0.0955703	newly 5 weeks spec	256.	339.02	{317., 360.}	32.4297	0.121404	newly 5 weeks cumul	3053.	3862.99	{3801., 3918.}	26.531	0.102158	newly 46 weeks cumul	3616.	4652.22	{4569., 4731.}	28.6565	0.109394
10	newly 3 weeks spec	32.	25.11	{15., 36.}	28.5938	0.155495	newly 3 weeks cumul	165.	173.02	{137., 206.}	13.2727	0.0555541	newly 5 weeks spec	7.	7.23	{1., 15.}	60.7143	0.360153	newly 5 weeks cumul	184.	194.04	{154., 238.}	14.7826	0.0615215	newly 50 weeks cumul	192.	201.68	{158., 248.}	15.4792	0.0648295
11	newly 3 weeks spec	582.	641.64	{602., 675.}	10.3952	0.0425241	newly 3 weeks cumul	2565.	2720.52	{2656., 2786.}	6.06316	0.0254906	newly 5 weeks spec	318.	332.44	{305., 355.}	6.47799	0.0271378	newly 5 weeks cumul	3307.	3514.99	{3430., 3607.}	6.28939	0.0264118	newly 44 weeks cumul	4044.	4378.95	{4268., 4479.}	8.28264	0.0344697
12	newly 3 weeks spec	488.	604.89	{574., 635.}	23.9529	0.0929041	newly 3 weeks cumul	2113.	2657.88	{2608., 2714.}	25.787	0.0995882	newly 5 weeks spec	223.	303.4	{279., 323.}	36.0538	0.132991	newly 5 weeks cumul	2630.	3387.73	{3329., 3458.}	28.811	0.109911	newly 45 weeks cumul	3083.	4113.67	{4037., 4197.}	33.4307	0.125212
13	newly 3 weeks spec	1177.	1194.2	{1156., 1239.}	2.38233	0.0101925	newly 3 weeks cumul	4644.	4795.24	{4723., 4870.}	3.25969	0.013897	newly 5 weeks spec	679.	689.6	{656., 721.}	3.3137	0.0141998	newly 5 weeks cumul	6187.	6395.34	{6324., 6485.}	3.36738	0.0143616	newly 43 weeks cumul	8302.	8570.33	{8474., 8656.}	3.23211	0.0137946
14	newly 3 weeks spec	9.	179.39	{161., 199.}	1893.22	1.29793	newly 3 weeks cumul	64.	953.75	{909., 1000.}	1390.23	1.17297	newly 5 weeks spec	5.	67.77	{56., 79.}	1255.4	1.12699	newly 5 weeks cumul	74.	1130.71	{1071., 1183.}	1427.99	1.18382	newly 48 weeks cumul	86.	1234.25	{1171., 1293.}	1335.17	1.15656
15	newly 3 weeks spec	73.	89.88	{75., 106.}	24.411	0.0922836	newly 3 weeks cumul	382.	497.68	{464., 532.}	30.2827	0.114224	newly 5 weeks spec	19.	32.06	{21., 44.}	70.3158	0.220711	newly 5 weeks cumul	449.	582.44	{546., 625.}	29.7194	0.112287	newly 48 weeks cumul	476.	626.51	{575., 675.}	31.6197	0.118374
16	newly 3 weeks spec	111.	262.49	{238., 287.}	136.477	0.372843	newly 3 weeks cumul	653.	1314.53	{1272., 1356.}	101.306	0.303718	newly 5 weeks spec	31.	106.98	{91., 124.}	245.097	0.535223	newly 5 weeks cumul	748.	1591.07	{1546., 1654.}	112.71	0.327654	newly 48 weeks cumul	812.	1787.03	{1734., 1850.}	120.078	0.342424
17	newly 3 weeks spec	848.	1006.75	{966., 1050.}	18.7205	0.0743152	newly 3 weeks cumul	3379.	4036.64	{3972., 4104.}	19.4626	0.0772012	newly 5 weeks spec	459.	582.25	{556., 604.}	26.8519	0.103011	newly 5 weeks cumul	4480.	5387.27	{5316., 5460.}	20.2516	0.0800695	newly 43 weeks cumul	5685.	7226.92	{7137., 7312.}	27.1226	0.104202
18	newly 3 weeks spec	371.	457.4	{421., 491.}	23.2884	0.0901954	newly 3 weeks cumul	1789.	2111.32	{2053., 2165.}	18.0168	0.0718443	newly 5 weeks spec	168.	211.52	{187., 235.}	25.9762	0.0985138	newly 5 weeks cumul	2207.	2633.58	{2559., 2705.}	19.3285	0.0766334	newly 44 weeks cumul	2601.	3069.27	{2976., 3177.}	18.0035	0.0717299
19	newly 3 weeks spec	522.	690.3	{652., 727.}	32.2414	0.121013	newly 3 weeks cumul	2300.	2966.71	{2910., 3021.}	28.9874	0.110499	newly 5 weeks spec	225.	359.87	{338., 386.}	59.9422	0.203481	newly 5 weeks cumul	2858.	3822.83	{3754., 3882.}	33.7589	0.126286	newly 46 weeks cumul	3344.	4741.92	{4661., 4817.}	41.8038	0.151646
20	newly 3 weeks spec	31.	100.62	{88., 115.}	224.581	0.508793	newly 3 weeks cumul	168.	567.36	{537., 601.}	237.714	0.52815	newly 5 weeks spec	4.	31.81	{23., 40.}	695.25	0.891932	newly 5 weeks cumul	185.	656.96	{622., 689.}	255.114	0.55002	newly 51 weeks cumul	187.	703.98	{664., 747.}	276.46	0.575341
21	newly 3 weeks spec	203.	260.79	{236., 279.}	28.468	0.107866	newly 3 weeks cumul	1027.	1311.33	{1260., 1358.}	27.6855	0.10598	newly 5 weeks spec	76.	106.02	{92., 118.}	39.5	0.142681	newly 5 weeks cumul	1233.	1587.89	{1536., 1648.}	28.7826	0.109707	newly 48 weeks cumul	1341.	1783.86	{1708., 1862.}	33.0246	0.123737
22	newly 3 weeks spec	138.	99.49	{84., 114.}	27.9058	0.144782	newly 3 weeks cumul	788.	561.71	{528., 598.}	28.717	0.147498	newly 5 weeks spec	76.	33.09	{25., 42.}	56.4605	0.370495	newly 5 weeks cumul	953.	652.11	{614., 692.}	31.5729	0.165279	newly 49 weeks cumul	1046.	699.18	{654., 744.}	33.1568	0.175525
23	newly 3 weeks spec	646.	910.94	{868., 948.}	41.0124	0.149021	newly 3 weeks cumul	2717.	3677.7	{3609., 3741.}	35.3589	0.131447	newly 5 weeks spec	343.	524.15	{495., 554.}	52.8134	0.183717	newly 5 weeks cumul	3563.	4895.12	{4825., 4960.}	37.3876	0.137922	newly 42 weeks cumul	4442.	6534.97	{6442., 6619.}	47.1177	0.167635
24	newly 3 weeks spec	888.	818.58	{781., 848.}	7.81757	0.0355852	newly 3 weeks cumul	3507.	3383.71	{3308., 3462.}	3.52866	0.0156604	newly 5 weeks spec	441.	444.55	{414., 479.}	4.33787	0.0186738	newly 5 weeks cumul	4589.	4431.48	{4351., 4518.}	3.43256	0.0152123	newly 42 weeks cumul	5895.	5685.57	{5588., 5812.}	3.55301	0.0157579
25	newly 3 weeks spec	135.	425.81	{310., 508.}	215.415	0.490584	newly 3 weeks cumul	801.	1928.83	{1519., 2191.}	140.803	0.376338	newly 5 weeks spec	46.	207.11	{140., 257.}	350.239	0.641367	newly 5 weeks cumul	936.	2432.82	{1882., 2782.}	159.917	0.40867	newly 45 weeks cumul	1018.	2895.68	{2170., 3352.}	184.448	0.446407
26	newly 3 weeks spec	1048.	1009.37	{968., 1047.}	3.88263	0.0173312	newly 3 weeks cumul	4173.	4041.12	{3984., 4110.}	3.16032	0.0139829	newly 5 weeks spec	644.	581.77	{549., 615.}	9.68789	0.0446583	newly 5 weeks cumul	5651.	5390.76	{5305., 5471.}	4.6052	0.0205049	newly 43 weeks cumul	7533.	7232.92	{7139., 7323.}	3.98354	0.0176782
27	newly 3 weeks spec	806.	660.17	{622., 694.}	18.0931	0.0870765	newly 3 weeks cumul	3332.	2757.24	{2695., 2809.}	17.2497	0.082292	newly 5 weeks spec	451.	354.01	{329., 377.}	21.5055	0.105768	newly 5 weeks cumul	4401.	3595.61	{3527., 3651.}	18.3002	0.087826	newly 44 weeks cumul	5811.	4570.29	{4460., 4650.}	21.3511	0.104369
28	newly 3 weeks spec	568.	414.27	{386., 443.}	27.0651	0.137681	newly 3 weeks cumul	2482.	1921.75	{1871., 1965.}	22.5725	0.111197	newly 5 weeks spec	266.	193.03	{171., 212.}	27.4323	0.140928	newly 5 weeks cumul	3132.	2398.22	{2343., 2467.}	23.4285	0.116036	newly 48 weeks cumul	3853.	2800.02	{2717., 2883.}	27.3288	0.138751
29	newly 3 weeks spec	769.	754.91	{712., 800.}	3.89727	0.0172117	newly 3 weeks cumul	3241.	3190.45	{3094., 3335.}	2.68467	0.0117726	newly 5 weeks spec	412.	399.08	{368., 441.}	6.37864	0.028413	newly 5 weeks cumul	4213.	4139.74	{4017., 4358.}	3.12509	0.0136965	newly 42 weeks cumul	5339.	5188.95	{4993., 5572.}	4.39314	0.0193759
30	newly 3 weeks spec	278.	266.21	{242., 287.}	5.7446	0.0260711	newly 3 weeks cumul	1314.	1339.53	{1297., 1376.}	2.67047	0.0114221	newly 5 weeks spec	87.	105.95	{91., 121.}	22.1494	0.0847836	newly 5 weeks cumul	1559.	1614.66	{1562., 1660.}	3.75625	0.0159296	newly 51 weeks cumul	1722.	1802.93	{1735., 1858.}	4.73229	0.0199556
31	newly 3 weeks spec	58.	19.1	{11., 26.}	67.069	0.503125	newly 3 weeks cumul	360.	146.02	{125., 165.}	59.4389	0.394674	newly 5 weeks spec	22.	4.68	{1., 8.}	78.7273	0.791954	newly 5 weeks cumul	413.	159.91	{135., 183.}	61.2809	0.415111	newly 52 weeks cumul	442.	164.01	{138., 189.}	62.8937	0.433886
32	newly 3 weeks spec	101.	51.86	{37., 66.}	48.6535	0.300436	newly 3 weeks cumul	549.	319.77	{278., 357.}	41.7541	0.236852	newly 5 weeks spec	30.	16.39	{8., 26.}	46.7667	0.316987	newly 5 weeks cumul	633.	365.3	{313., 411.}	42.2907	0.241298	newly 49 weeks cumul	682.	383.45	{318., 438.}	43.7757	0.2531
33	newly 3 weeks spec	918.	753.64	{717., 794.}	17.9041	0.0860035	newly 3 weeks cumul	3832.	3259.75	{3194., 3316.}	14.9335	0.0702804	newly 5 weeks spec	479.	384.86	{359., 412.}	19.6534	0.0957825	newly 5 weeks cumul	4959.	4186.02	{4117., 4258.}	15.5874	0.0736296	newly 47 weeks cumul	6261.	5145.4	{5051., 5233.}	17.8182	0.0852722
34	newly 3 weeks spec	843.	1002.17	{963., 1039.}	18.8814	0.0748897	newly 3 weeks cumul	3518.	4044.19	{3956., 4123.}	14.9571	0.0604855	newly 5 weeks spec	471.	578.64	{547., 610.}	22.8535	0.0890112	newly 5 weeks cumul	4638.	5394.29	{5290., 5491.}	16.3064	0.0655567	newly 42 weeks cumul	6028.	7180.65	{7030., 7335.}	19.1216	0.0759361
35	newly 3 weeks spec	693.	535.25	{499., 567.}	22.7633	0.112734	newly 3 weeks cumul	3121.	2448.46	{2398., 2501.}	21.5489	0.105459	newly 5 weeks spec	338.	253.07	{226., 281.}	25.1272	0.127051	newly 5 weeks cumul	3977.	3069.68	{2995., 3135.}	22.8142	0.112527	newly 47 weeks cumul	4760.	3610.8	{3526., 3702.}	24.1429	0.120093
36	newly 3 weeks spec	635.	752.04	{720., 787.}	18.4315	0.073218	newly 3 weeks cumul	2904.	3171.36	{3122., 3224.}	9.20661	0.0382103	newly 5 weeks spec	364.	401.45	{373., 430.}	10.3599	0.0422331	newly 5 weeks cumul	3816.	4121.42	{4048., 4206.}	8.00367	0.0333933	newly 45 weeks cumul	4844.	5195.64	{5069., 5310.}	7.25929	0.0303661
37	newly 3 weeks spec	121.	95.85	{80., 110.}	20.9008	0.104956	newly 3 weeks cumul	634.	544.64	{510., 581.}	14.0946	0.0664804	newly 5 weeks spec	49.	30.53	{20., 40.}	37.7755	0.218321	newly 5 weeks cumul	759.	630.24	{589., 677.}	16.9644	0.0813684	newly 50 weeks cumul	824.	670.21	{619., 720.}	18.6638	0.0904577
38	newly 3 weeks spec	12.	32.39	{23., 42.}	169.917	0.416147	newly 3 weeks cumul	113.	211.88	{183., 240.}	87.5044	0.27036	newly 5 weeks spec	3.	8.58	{3., 14.}	196.	0.46227	newly 5 weeks cumul	121.	237.11	{199., 276.}	95.9587	0.288995	newly 50 weeks cumul	126.	245.63	{205., 292.}	94.9444	0.28648
39	newly 3 weeks spec	302.	448.43	{413., 481.}	48.4868	0.170905	newly 3 weeks cumul	1471.	2018.12	{1937., 2093.}	37.1937	0.13718	newly 5 weeks spec	115.	212.63	{194., 236.}	84.8957	0.26544	newly 5 weeks cumul	1796.	2537.58	{2459., 2628.}	41.2906	0.149963	newly 44 weeks cumul	2002.	3005.73	{2884., 3132.}	50.1364	0.176282
40	newly 3 weeks spec	529.	571.96	{534., 608.}	8.40076	0.034601	newly 3 weeks cumul	2325.	2434.49	{2383., 2498.}	4.7514	0.0200648	newly 5 weeks spec	275.	299.84	{275., 323.}	9.55636	0.0390469	newly 5 weeks cumul	2963.	3149.7	{3081., 3212.}	6.32332	0.0265451	newly 45 weeks cumul	3697.	3930.39	{3839., 4015.}	6.32594	0.0265519
41	newly 3 weeks spec	253.	416.68	{387., 447.}	64.6957	0.216085	newly 3 weeks cumul	1228.	1871.9	{1808., 1934.}	52.4349	0.182938	newly 5 weeks spec	114.	196.06	{173., 219.}	71.9825	0.233707	newly 5 weeks cumul	1511.	2355.67	{2277., 2421.}	55.9014	0.192719	newly 46 weeks cumul	1685.	2797.28	{2682., 2906.}	66.0107	0.219944
42	newly 3 weeks spec	23.	25.99	{18., 36.}	26.3913	0.104058	newly 3 weeks cumul	166.	168.32	{144., 197.}	10.3855	0.0446903	newly 5 weeks spec	8.	7.23	{2., 14.}	48.625	0.300459	newly 5 weeks cumul	186.	190.17	{153., 229.}	11.7473	0.0501185	newly 50 weeks cumul	187.	198.36	{155., 237.}	13.6791	0.0563831
43	newly 3 weeks spec	681.	819.35	{783., 849.}	20.3157	0.0800811	newly 3 weeks cumul	3118.	3482.16	{3413., 3545.}	11.6793	0.0479288	newly 5 weeks spec	377.	436.48	{402., 465.}	15.7772	0.0629805	newly 5 weeks cumul	4043.	4513.35	{4452., 4573.}	11.6337	0.0477656	newly 43 weeks cumul	5018.	5655.95	{5546., 5757.}	12.7132	0.0519294
44	newly 3 weeks spec	174.	270.78	{252., 293.}	55.6207	0.191358	newly 3 weeks cumul	824.	1303.85	{1270., 1337.}	58.2342	0.19919	newly 5 weeks spec	61.	115.77	{100., 128.}	89.7869	0.276268	newly 5 weeks cumul	988.	1597.67	{1551., 1642.}	61.7075	0.208623	newly 49 weeks cumul	1084.	1824.08	{1767., 1873.}	68.2731	0.2259
45	newly 3 weeks spec	45.	89.03	{74., 104.}	97.8444	0.292391	newly 3 weeks cumul	244.	493.52	{459., 529.}	102.262	0.305134	newly 5 weeks spec	9.	30.26	{21., 40.}	236.222	0.512761	newly 5 weeks cumul	276.	576.19	{525., 615.}	108.764	0.318854	newly 48 weeks cumul	280.	619.87	{560., 667.}	121.382	0.344186
46	newly 3 weeks spec	223.	323.48	{297., 344.}	45.0583	0.160812	newly 3 weeks cumul	1040.	1618.64	{1572., 1670.}	55.6385	0.191998	newly 5 weeks spec	61.	136.42	{118., 153.}	123.639	0.347447	newly 5 weeks cumul	1223.	1966.25	{1911., 2021.}	60.7727	0.206096	newly 48 weeks cumul	1322.	2206.58	{2138., 2271.}	66.9123	0.222347
47	newly 3 weeks spec	132.	95.88	{81., 112.}	27.3636	0.142403	newly 3 weeks cumul	614.	547.5	{510., 582.}	10.8306	0.0503868	newly 5 weeks spec	43.	31.56	{23., 41.}	27.2558	0.147258	newly 5 weeks cumul	740.	634.6	{589., 674.}	14.2432	0.0673427	newly 50 weeks cumul	804.	678.97	{626., 722.}	15.551	0.0740589
48	newly 3 weeks spec	186.	151.77	{135., 170.}	18.4032	0.0901339	newly 3 weeks cumul	969.	837.22	{806., 869.}	13.5996	0.0636661	newly 5 weeks spec	49.	55.69	{45., 66.}	16.7143	0.065515	newly 5 weeks cumul	1115.	986.11	{948., 1020.}	11.5596	0.053526	newly 50 weeks cumul	1182.	1068.66	{1020., 1114.}	9.58883	0.0439794
49	newly 3 weeks spec	8.	26.43	{18., 35.}	230.375	0.50701	newly 3 weeks cumul	68.	173.27	{156., 189.}	154.809	0.4048	newly 5 weeks spec	3.	7.26	{4., 11.}	143.333	0.350064	newly 5 weeks cumul	72.	194.21	{173., 213.}	169.736	0.429354	newly 52 weeks cumul	76.	202.01	{178., 226.}	165.803	0.422724
50	newly 3 weeks spec	733.	689.26	{658., 720.}	6.12278	0.0276984	newly 3 weeks cumul	2944.	2872.35	{2806., 2931.}	2.55673	0.0113191	newly 5 weeks spec	408.	372.2	{340., 401.}	8.81373	0.0407566	newly 5 weeks cumul	3889.	3749.6	{3683., 3818.}	3.5927	0.0159662	newly 43 weeks cumul	4985.	4781.9	{4710., 4883.}	4.07422	0.0181352
